# Supplementary material for: Unveiling the Bioactive Potential of the Invasive Jellyfish Phyllorhiza punctata Through Integrative Transcriptomic and Proteomic Analyses
Source: Biomolecules. 2025 Aug 4;15(8):1121. doi: 10.3390/biom15081121 (PMC12383608; doi:10.3390/biom15081121)
Supplement: Supplementary file 1 [file biomolecules-15-01121-s001.zip › Suplementary Material/FileS3/PEPFOLD4/XP_065058710_9575/clusters.html]

 PEP-FOLD clusters


Download archive of all models  here   

|  |  |  |
| --- | --- | --- |
| # | file | sOPEP |
| # cluster 1 - Size: 11 |  |  |
|  | XP\_065058710\_9575-model1 | -10.0261 |
|  | XP\_065058710\_9575-model1.1 | -9.75497 |
|  | XP\_065058710\_9575-model1.2 | -9.52033 |
|  | XP\_065058710\_9575-model1.3 | -9.52033 |
|  | XP\_065058710\_9575-model1.4 | -9.52033 |
|  | XP\_065058710\_9575-model1.5 | -9.52033 |
|  | XP\_065058710\_9575-model1.6 | -9.458 |
|  | XP\_065058710\_9575-model1.7 | -9.458 |
|  | XP\_065058710\_9575-model1.8 | -9.38112 |
|  | XP\_065058710\_9575-model1.9 | -9.08296 |
|  | XP\_065058710\_9575-model1.10 | -9.08296 |
| # cluster 2 - Size: 43 |  |  |
|  | XP\_065058710\_9575-model2 | -9.09841 |
|  | XP\_065058710\_9575-model2.1 | -9.09237 |
|  | XP\_065058710\_9575-model2.2 | -9.09237 |
|  | XP\_065058710\_9575-model2.3 | -9.09237 |
|  | XP\_065058710\_9575-model2.4 | -9.07727 |
|  | XP\_065058710\_9575-model2.5 | -9.07727 |
|  | XP\_065058710\_9575-model2.6 | -9.07727 |
|  | XP\_065058710\_9575-model2.7 | -9.07727 |
|  | XP\_065058710\_9575-model2.8 | -9.07727 |
|  | XP\_065058710\_9575-model2.9 | -9.07727 |
|  | XP\_065058710\_9575-model2.10 | -9.07727 |
|  | XP\_065058710\_9575-model2.11 | -9.07727 |
|  | XP\_065058710\_9575-model2.12 | -9.07727 |
|  | XP\_065058710\_9575-model2.13 | -9.07727 |
|  | XP\_065058710\_9575-model2.14 | -9.06678 |
|  | XP\_065058710\_9575-model2.15 | -9.0602 |
|  | XP\_065058710\_9575-model2.16 | -9.0602 |
|  | XP\_065058710\_9575-model2.17 | -9.0602 |
|  | XP\_065058710\_9575-model2.18 | -9.0602 |
|  | XP\_065058710\_9575-model2.19 | -9.02851 |
|  | XP\_065058710\_9575-model2.20 | -9.02851 |
|  | XP\_065058710\_9575-model2.21 | -9.02851 |
|  | XP\_065058710\_9575-model2.22 | -9.02851 |
|  | XP\_065058710\_9575-model2.23 | -9.02851 |
|  | XP\_065058710\_9575-model2.24 | -9.02851 |
|  | XP\_065058710\_9575-model2.25 | -9.02491 |
|  | XP\_065058710\_9575-model2.26 | -9.01276 |
|  | XP\_065058710\_9575-model2.27 | -9.01276 |
|  | XP\_065058710\_9575-model2.28 | -9.01276 |
|  | XP\_065058710\_9575-model2.29 | -9.01041 |
|  | XP\_065058710\_9575-model2.30 | -9.00955 |
|  | XP\_065058710\_9575-model2.31 | -9.0062 |
|  | XP\_065058710\_9575-model2.32 | -8.99309 |
|  | XP\_065058710\_9575-model2.33 | -8.9256 |
|  | XP\_065058710\_9575-model2.34 | -8.9182 |
|  | XP\_065058710\_9575-model2.35 | -8.91719 |
|  | XP\_065058710\_9575-model2.36 | -8.91719 |
|  | XP\_065058710\_9575-model2.37 | -8.90412 |
|  | XP\_065058710\_9575-model2.38 | -8.86397 |
|  | XP\_065058710\_9575-model2.39 | -8.84632 |
|  | XP\_065058710\_9575-model2.40 | -8.8337 |
|  | XP\_065058710\_9575-model2.41 | -8.82972 |
|  | XP\_065058710\_9575-model2.42 | -8.7316 |
| # cluster 3 - Size: 4 |  |  |
|  | XP\_065058710\_9575-model3 | -9.09188 |
|  | XP\_065058710\_9575-model3.1 | -8.90583 |
|  | XP\_065058710\_9575-model3.2 | -8.90583 |
|  | XP\_065058710\_9575-model3.3 | -8.85861 |
| # cluster 4 - Size: 42 |  |  |
|  | XP\_065058710\_9575-model4 | -9.04197 |
|  | XP\_065058710\_9575-model4.1 | -9.01022 |
|  | XP\_065058710\_9575-model4.2 | -9.01022 |
|  | XP\_065058710\_9575-model4.3 | -9.01022 |
|  | XP\_065058710\_9575-model4.4 | -9.01022 |
|  | XP\_065058710\_9575-model4.5 | -8.99371 |
|  | XP\_065058710\_9575-model4.6 | -8.96574 |
|  | XP\_065058710\_9575-model4.7 | -8.96471 |
|  | XP\_065058710\_9575-model4.8 | -8.96273 |
|  | XP\_065058710\_9575-model4.9 | -8.96273 |
|  | XP\_065058710\_9575-model4.10 | -8.96249 |
|  | XP\_065058710\_9575-model4.11 | -8.96249 |
|  | XP\_065058710\_9575-model4.12 | -8.95284 |
|  | XP\_065058710\_9575-model4.13 | -8.95284 |
|  | XP\_065058710\_9575-model4.14 | -8.95284 |
|  | XP\_065058710\_9575-model4.15 | -8.95284 |
|  | XP\_065058710\_9575-model4.16 | -8.91347 |
|  | XP\_065058710\_9575-model4.17 | -8.87394 |
|  | XP\_065058710\_9575-model4.18 | -8.8699 |
|  | XP\_065058710\_9575-model4.19 | -8.8699 |
|  | XP\_065058710\_9575-model4.20 | -8.8699 |
|  | XP\_065058710\_9575-model4.21 | -8.86969 |
|  | XP\_065058710\_9575-model4.22 | -8.86969 |
|  | XP\_065058710\_9575-model4.23 | -8.86969 |
|  | XP\_065058710\_9575-model4.24 | -8.86969 |
|  | XP\_065058710\_9575-model4.25 | -8.86825 |
|  | XP\_065058710\_9575-model4.26 | -8.8278 |
|  | XP\_065058710\_9575-model4.27 | -8.81729 |
|  | XP\_065058710\_9575-model4.28 | -8.81527 |
|  | XP\_065058710\_9575-model4.29 | -8.81527 |
|  | XP\_065058710\_9575-model4.30 | -8.81527 |
|  | XP\_065058710\_9575-model4.31 | -8.81527 |
|  | XP\_065058710\_9575-model4.32 | -8.81527 |
|  | XP\_065058710\_9575-model4.33 | -8.81261 |
|  | XP\_065058710\_9575-model4.34 | -8.80972 |
|  | XP\_065058710\_9575-model4.35 | -8.79751 |
|  | XP\_065058710\_9575-model4.36 | -8.78658 |
|  | XP\_065058710\_9575-model4.37 | -8.76688 |
|  | XP\_065058710\_9575-model4.38 | -8.76688 |
|  | XP\_065058710\_9575-model4.39 | -8.75678 |
|  | XP\_065058710\_9575-model4.40 | -8.73222 |
|  | XP\_065058710\_9575-model4.41 | -8.70752 |
|  |  |  |
